# On the adequacy of static analysis warnings with respect to code smell prediction

**DOI:** 10.1007/s10664-022-10126-5

**Published:** 2022-03-17

**Authors:** Fabiano Pecorelli, Savanna Lujan, Valentina Lenarduzzi, Fabio Palomba, Andrea De Lucia

**Affiliations:** 1 Tampere University, Tampere, Finland; 2 LUT University, Lappeenranta, Finland; 3 University of Oulu, Oulu, Finland; 4 SeSa Lab, University of Salerno, Fisciano, Italy

**Keywords:** Code Smells, Static Analysis Tools, Machine Learning.

## Abstract

Code smells are poor implementation choices that developers apply while evolving source code and that affect program maintainability. Multiple automated code smell detectors have been proposed: while most of them relied on heuristics applied over software metrics, a recent trend concerns the definition of machine learning techniques. However, machine learning-based code smell detectors still suffer from low accuracy: one of the causes is the lack of adequate features to feed machine learners. In this paper, we face this issue by investigating the role of static analysis warnings generated by three state-of-the-art tools to be used as features of machine learning models for the detection of seven code smell types. We conduct a three-step study in which we (1) verify the relation between static analysis warnings and code smells and the potential predictive power of these warnings; (2) build code smell prediction models exploiting and combining the most relevant features coming from the first analysis; (3) compare and combine the performance of the best code smell prediction model with the one achieved by a state of the art approach. The results reveal the low performance of the models exploiting static analysis warnings alone, while we observe significant improvements when combining the warnings with additional code metrics. Nonetheless, we still find that the best model does not perform better than a random model, hence leaving open the challenges related to the definition of ad-hoc features for code smell prediction.

## Introduction

Software maintenance is known to be the most expensive phase of the software lifecycle (Banker et al. 1993). This is not only due to continuous change requests, but also to the increasing complexity that make developers unable to cope with software quality requirements (Lehman 1996). Indeed, in this scenario developers are often enforced to set aside good design and implementation principles in order to deliver fast, possibly letting emerge the so-called *technical debt* (Cunningham 1992), i.e., the introduction of quick workarounds in the source code that worsen its maintainability.

A relevant form of technical debt is represented by *bad code smells* (Fowler and Beck 1999), a.k.a., *code smells* or simply *smells*: these are symptoms of poor implementation solutions that previous research has negatively related to program comprehensibility (Abbes et al. 2011; Politowski et al. 2020), change- and defect-proneness (Khomh et al. 2012; Palomba et al. 2018b), and maintenance costs (Sjøberg et al. 2012; Soh et al. 2016). The previous empirical investigations into the relation between code smells and software maintainability has motivated researchers in defining automated solutions for detecting code smells (Azeem et al. 2019; de Paulo Sobrinho et al. 2018).

Most of the existing techniques rely on the combination of various software metrics (e.g., cohesion and coupling (Chidamber and Kemerer 1994)) through rules and heuristics (Moha et al. 2009; Palomba et al. 2014b; Palomba et al. 2016)). While these have been shown to reach an acceptable accuracy, there are still some key limitations that preclude their wide usage in practice. In the first place, the output of these heuristic-based detectors cannot be objectively assessed by developers (Arcelli Fontana et al. 2016a; Palomba et al. 2014a; Taibi et al. 2017). Secondly, different detectors do not output the same results, making even harder for developers to decide on whether to refactor source code (Arcelli Fontana et al. 2012). Finally, these detectors require thresholds to distinguish smelly from non-smelly components which are hard to tune (Arcelli et al. 2015).

For the above-mentioned reasons, researchers have been starting considering the application of machine learning techniques as an alternative. Indeed, these may be exploited to address the limitations of heuristic methods by combining multiple metrics and learning code smell instances considered relevant by developers without the specification of any threshold (Azeem et al. 2019). Nonetheless, the promises of machine learning-based code smell detection have not yet been kept. Di Nucci et al. (2018b) showed that these detectors fail in most cases, while (Pecorelli et al. 2019; Pecorelli et al. 2020a) identified (1) the little contributions given by the features investigated so far and (2) the limited amount of code smell instances available to train a machine learner in an appropriate manner as the two main causes leading to those failures.

In this article, we started addressing the first problem by conducting a preliminary investigation into the contribution given by the warnings of automated static analysis tools to the classification capabilities of machine learning-based code smell detectors. The choice of focusing on those warnings was motivated by the type of design issues that can be identified through static analysis tools. More particularly, while some of the warnings they raise are not directly related to source code design and code quality, there are several exceptions. For instance, let consider the warning category called *‘bad_practice’* raised by FindBugs, one of the most widely used static analysis tools in practice (Vassallo et al. 2019). According to the list of warnings reported in the official documentation,^1^ this category includes a number of design-related warnings. Similarly, the warning category *‘design’* provided by Checkstyle and PMD is also associated with design issues. As such, static analysis tools actually deal with the design of source code and pinpoint a number of violations that may be connected to the presence of code smells. In the context of this paper, we first hypothesized that the indications provided by the static analysis tools (Wedyan et al. 2009) can be potentially useful to characterize code smell instances. Secondly, we conjectured that the incorporation of these warnings within intelligent systems may represent a way to reduce the high amount of false positives they output (Johnson et al. 2013).

To verify our hypotheses, we have investigated the potential contribution given by individual types of warnings output by three static analysis tools, i.e., Checkstyle, FindBugs, and PMD, to the prediction of three code smell types, i.e., *God Class*, *Spaghetti Code*, and *Complex Class*. To this purpose, we analyzed five open-source projects. Then, we used the most relevant features coming from the first analysis to build and assess the capabilities of machine learning models when detecting the three considered smells. The results of the study highlighted promising results: models built using the warnings of individual static analysis tools score between 55% and 91% in terms of F-Measure, while the warning types that contribute the most to the performance of the learners depended on the specific code smell considered.

This paper extends our previous work (Lujan et al. 2020a) and enlarges our investigation toward the usefulness of static analysis warnings for machine learning-based code smell detection. We extend the number of code smells and software projects considered, taking into account a total of seven code smell types over 25 releases of 5 open-source projects. Afterwards, we design a three-step empirical study. First, we conduct a preliminary, motivational investigation into the actual relation between static analysis warnings and code smells, also attempting to assess the potential predictive power of those warnings.

Second, we start replicating the study conducted in our original paper (Lujan et al. 2020a), analyzing the performance of code smell detection techniques based machine learners and using the static analysis warnings as features. The results of our replication study **do not** confirm our previous findings: indeed, when considering a larger set of projects, the performance of the machine learners are way lower, especially in terms of precision. In response to this negative result, we further investigate the problem by studying the overlap among the predictions made by machine learning models built using the warnings of different static analysis tools as features: such an analysis reveals a high complementarity suggesting that a combination of those warnings could potentially improve the code smell detection capabilities. As such, we define and experiment a new combined model which significantly perform better than the individual models. In the last part of our study, we go beyond and analyze how this combined model can be further combined with additional code metrics that have been used for code smell detection in previous work (Azeem et al. 2019). While the performance of the combined model significantly performs better than previous approaches based on software metrics.

To sum up, our paper provides the following contributions:

1. A preliminary analysis on the suitability of static analysis warnings in the context of code smell detection;
2. An empirical understanding of how machine learning techniques for code smell detection work when fed with warnings generated by automated static analysis tools;
3. A machine learning-based detector that combines multiple automated static analysis tools, improving on the performance of individual detectors;
4. An empirical understanding of how warning-based machine learning techniques for code smell detection work in comparison with metric-based ones;
5. A machine learning-based detector that combines static analysis warnings and code metrics, further improving detectors’ performance;
6. A comprehensive replication package (Pecorelli et al. 2021) which reports all data used in our study and that can be used by researchers to verify/replicate our results as well as build upon our findings.

### Structure of the Paper

Section 2 overviews the state of the art in machine learning for code smell detection. Section 3 reports the methodology employed to address our research objectives, while Section 4 reports the results obtained. Section 5 further discusses the main findings of the study and overviews the implications that they have for the research community. In Section 6 we discuss the threats to the validity of our study. Finally, Section 7 concludes the paper and discusses our future research agenda.

## Related Work

The use of machine learning techniques for code smell detection is recently gaining attention, as proved by the amount of publications in the last years. The interested reader can find a complete overview of the research done in the field in the survey by Azeem et al. (2019).

### Machine Learning for Code Smell Detection

Some early work has been conducted with the aim of devising machine learning solutions that could be applied to detect individual code smell types, e.g., White et al. (2016), Khomh et al. (2009), and Khomh et al. (2011). More recent papers have instead attempted to make machine learning techniques general enough to support the identification of multiple code smells. This is clearly the case of our empirical study and, for this reason, we overview in the following the papers more closely connected.

Kreimer (2005) proposed a detection approach for two code smells (Long Method and Large Class) based on a decision tree model in two software systems. The model provided a good level of accuracy. The achieved results were later confirmed by Amorin et al. (Amorim et al. 2015), who tested the previous technique over a medium-scale system, reaching an accuracy up to 78%.

Khomh et al. (2011) and Khomh et al. (2009) employed Bayesian belief networks for the detection of three code smells (Blob, Functional Decomposition, and Spaghetti Code) from different open-source software, obtaining promising results.

Maiga et al. (2012b) adopted a support vector machine based approach to build a code smell detection model. The model was trained using software metrics as features for each instance and was extended taking into account the practitioners feedback (Maiga et al. 2012a). The extended model is able to capture four code smells (Blob, Functional Decomposition, Spaghetti Code and Swiss Army Knife) with an accuracy up to 74%.

Arcelli Fontana et al. were among the most active researchers in the field and applied machine learning techniques to detect multiple code smell types (Arcelli Fontana et al. 2016b), estimate their harmfulness (Arcelli Fontana et al. 2016b), and compute their intensity (Arcelli Fontana and Zanoni 2017), showing the potential usefulness of these techniques. More specifically, in (Arcelli Fontana et al. 2016b) they applied 16 different machine-learning techniques on four types of code smells (Data Class, Large Class, Feature Envy, Long Method) and on 74 software systems. The highest accuracy (up to 95%) was achieved by J48 and Random Forest. In a follow-up study (Arcelli Fontana and Zanoni 2017), the authors focused on the classification of these four code smell severity using the same machine learning techniques. Also in this work, the best models reached highest accuracy level (88%–96%).

In a replication study conducted by Di Nucci et al. (2018b), the authors pointed out that the accuracy of machine learning-based code smell detectors is strongly connected to the reliability of the dependent variable. This study has driven our choice of focusing on a manually-built and publicly available dataset of code smell instances (Palomba et al. 2018a; Palomba et al. 2015).

Pecorelli et al. (2020b) investigated the adoption of machine learning to classify code smells based on the perceived criticality. The authors ranked four code smells (God Class, Complex Class, Spaghetti Code, and Shotgun Surgery) based on machine learning depending on the harmfulness assigned by developers. Results showed that Random forest was the best modelling technique with an accuracy between 72% and 85%. Pecorelli et al. (2019) and Pecorelli et al. (2020a) also focused on the role of data balancing for code smell prediction. More particularly, the authors first conducted a large-scale study to compare the performance of heuristic-based and machine learning techniques (Random Forest, J48, Support Vector Machine, and Naıve Bayes algorithm) using metrics to detect five code smells (God Class, Spaghetti Cod, Class Data Should be Private, Complex Class, and Long Method) in 25 releases of 13 software systems (Pecorelli et al. 2019): their results revealed that heuristic-based technique has a slightly better performance than machine learning approaches and that one of the key issues making the performance of machine learning poor was the high imbalance between smelly and non-smelly components arising in real software systems. In a follow-up work (Pecorelli et al. 2020a), the authors discovered that, in most cases, machine learning-based detectors work better when no balancing is applied.

A recent study (Shcherban et al. 2020) applied two machine learning algorithms (Logistic Regression and Bag of Words) to better locate code smells with a precision of 98% and a recall of 97%. Differently from the others, this approach mines and analyzes code smell discussions from textual artefacts (e.g., code reviews).

The role of machine learning algorithms was also investigated in the context of the relation between code quality and fault prediction capabilities (Ma et al. 2016; Palomba et al. 2017). Finally, Lujan et al. (Lujan et al. 2020b) investigated the possibility of prioritizing code smell refactoring with the help of fault prediction results.

With respect to the papers discussed above, ours must be seen as complementary. We aimed at assessing the capabilities of the warnings raised by automated static analysis tools as features for code smell prediction. As such, we build upon the literature on the identification of proper features for detecting code smells and present a novel methodology.

### Machine Learning for Static Analysis Tools Detection

On a different note, a few works have applied machine learning techniques to analyze static analysis warnings and, particularly, to evaluate change- and fault-proneness of SonarQube violations (I Tollin et al. 2017; Falessi et al. 2017; Lenarduzzi et al. 2019a).

I Tollin et al. (2017), analyzed in the context of two industrial projects, analyzed whether the warnings given by the tool are associated to classes with higher change-proneness, confirming the relation. Falessi et al. (Falessi et al. 2017) analyzed 106 SonarQube violations in an industrial project: the results demonstrated that 20% of faults were preventable should these violations have been removed.

Lenarduzzi et al. (2019a) assessed the fault-proneness of SonarQube violations on 21 open-source systems applying seven machine learning algorithms (AdaBoost, Bagging, Decision Tree, Extremely Randomized Trees, Gradient Boosting, Random Forest, and XGBoost), and logistic regression. Results showed that violations classified as *“bugs”* hardly lead to a failure.

Another work (Lenarduzzi et al. 2019b) applied eight machine learning techniques (Linear Regression, Random Forest, Gradient Boost, Extra Trees, Decision Trees, Bagging, AdaBoost, SVM) on 33 Java projects, to understand if Technical Debt—based on SonarQube violations—could be derived from the 28 software metrics measured by SonarQube. Results show that technical debt are not correlated with the 28 software metrics. Considering another static analysis tool, a recent study (Lenarduzzi et al. 2021) investigated if pull requests are accepted in open-source based on quality flaws identified by PMD. The study considered 28 Java open-source projects, analyzing the presence of 4.7M PMD rules in 36K pull requests. As machine Learning, they used eight different classifiers: Logistic Regression, AdaBoost, Bagging, Decision Tree, ExtraTrees, GradientBoost, Random Forest, and XGBoost. Unexpectedly, quality flaws measured by PMD turned out not to affect the acceptance of a pull request at all.

Our work is complementary to those discussed above, since our goal is to exploit the outcome of different static analysis tools in order to improve the accuracy of code smell detection.

## Research Methodology

In the context of this empirical study, we had the ultimate goal of assessing the extent to which static analysis warnings can contribute to the identification of design issues in source code. We faced this goal by means of multiple analyses and research angles.

We defined three main dimensions. At first, we conducted a statistical study aiming at investigating whether and to what extent can static analysis warnings be actually used and useful in the context of code smell detection. Such an analysis must be deemed as preliminary, since it allowed us to quantify the potential benefits provided by those warnings: should this have not provided sufficiently acceptable results, this would have already stopped our investigation. On the contrary, a positive result would have provided further motivations into the need for a closer investigation on the role of static analysis warnings for code smell detection.

In this regard, we defined the first two research questions. In the first place, we aimed at assessing if the distribution of static analysis warnings differs when computed on classes affected and not affected by code smells. Rather than approaching the problem from a correlation perspective, we preferred to use a distribution analysis since the latter may provide insights on the specific types of warnings that are statistically different in the two sets of classes, i.e., smelly or smelly-free—on the contrary, correlations might have only given an indication of the strength of association, without reporting on the statistical significance when computed on smelly and non-smelly classes. We asked:

### RQ_1_

 How do static analysis warning types differ in classes affected and not affected by code smells?

In the second place, we complemented the distribution analysis with an additional investigation into the potential usefulness of static analysis warnings for code smell detection. While the first preliminary analysis had the goal to assess the distribution of warnings in classes affected or not by code smells, this second step aimed at quantifying the contribution that such warnings might provide to code smell prediction models. In particular, we asked:

### RQ_2_

 How do static analysis warnings contribute to the classification of code smells?

Once we had ensured the feasibility of a deeper analysis, we then proceeded with the investigation of the performance achieved by a code smell detection model relying on static analysis warnings as predictors. This analysis allowed us to provide quantitative insights on the actual usefulness of static analysis warnings, other than understanding their limitations when considered in the context of code smell detection. This led to the definition of three additional research questions.

First, on the basis of the results achieved in the preliminary study, we devised machine learning-based techniques—one for each static analysis tool considered, as explained later in this section—that exploit the warnings providing more contribution to the classification of code smells. Afterwards, we assessed their performance by addressing **RQ**_3_:

### RQ_3_

 How do machine learning techniques that exploit the warnings of single static analysis tools perform in the context of code smell detection?

Once we had assessed the classification performance of the individual models created in **RQ**_3_, we discovered that these models had low performance, especially due to false positives. To overcome this issue, we moved toward the analysis of the complementarity between the individual models, namely the extent to which different models could identify different code smell instances. This was relevant because a positive answer could have paved the way to a combination of multiple models. Hence, we asked:

### RQ_4_

 What is the orthogonality among the individual machine learning-based code smell detectors?

Given the results achieved when addressing **RQ**_4_, we then devised a combined model. The process required the identification of the optimal subset of the static analysis warnings exploited by different tools. While investigating the performance of such a combined model, we addressed **RQ**_5_:

### RQ_5_

 How do machine learning techniques that combine the warnings of different static analysis tools perform in the context of code smell detection?

The analyses defined so far could help understand how static analysis warnings enable the identification of code smells. Yet, it is important to remark that the research on machine learning for code smell detection has been vibrant over the last years (Azeem et al. 2019) and, as a matter of fact, a number of researchers has been working on the optimization of machine learning pipelines with the goal of improving the code smell detection capabilities. We took into account this aspect when defining the third part of our investigation. The last part of the empirical study consisted of the definition of the last three research questions.

First, we compared the best machine learner coming from the previous study, namely the one that combines the static analysis warnings coming from different tools, with a machine learner that exploits structural code metrics, namely a state of the art solution that has been used multiple times in the past (Azeem et al. 2019). This led to the formulation of our **RQ**_6_:

### RQ_6_

 How does the combined machine learner work when compared to an existing, code metrics-based approach for code smell detection?

Afterwards, we proceeded with a complementarity analysis involving the two techniques (i.e., the combined machine learner and the metrics-based approach for code smell detection) in order to understand to what extent the models built on two different sets of metrics could identify identify different code smell instances. In case of a positive answer, better performance could be achieved by combining these two sets of metrics together. In this regard, we asked the following research question:

### RQ_7_

 What is the orthogonality among the combined machine learner and the metrics-based approach for code smell detection?

Finally, after we have studied the complementarity between the two models, we evaluated an additional combination, which aimed at putting together static analysis warnings and code metrics. Hence, we asked:

### RQ_8_

 How do machine learning techniques that combine static analysis warnings and code metrics perform in the context of code smell detection?

The next sections report on the data selection, collection, and analysis procedures adopted to address our research questions.

### Context of the Study

The *context* of the study was composed of open-source software projects, code smells, and static analysis tools.

#### Selection of Code Smells

The exploited dataset reports code smell instances pertaining to 13 different types. However, not all of them are suitable for a machine learning solution. For instance, let consider the case of *Class Data Should Be Private*: this smell appears when a class exposes its attributes, i.e., the attributes have a public visibility. By definition, instances of this code smell can be effectively detected using simpler rule-based mechanisms, as done in the past (Moha et al. 2009).

For this reason, we first filtered out the code smell types whose definitions do not require any threshold. In addition, we filtered out method-level code smells, e.g., *Long Method*. The decision was driven by three main observations. In the first place, the vast majority of the previous papers on code smell prediction have used a class-level granularity (Azeem et al. 2019) and, therefore, our choice allowed for a simpler interpretation and comparison of the results. Secondly, our study focuses on the code smells perceived by developers as the most harmful (Taibi et al. 2017; Palomba et al. 2014a), which are all at class-level. Thirdly, the analyses performed in the context of our empirical study required the use of a heuristic code smell detector (i.e., Decor (Moha et al. 2009)) that has been designed and experimentally tested on class-level code smells. All these reasons led us to conclude that considering method-level code smells would not be necessarily beneficial for the paper. Nonetheless, our future research on the matter will consider the problem of assessing the role of static analysis warnings for the detection of method-level code smells.

Based on these considerations, we focused our study on the following seven code smells:

- **God Class.** Also known as *Blob*, this smell generally appears when a class is large, poorly cohesive, and has a number of dependencies with other data classes of the system (Fowler and Beck 1999).
- **Spaghetti Code.** Instances of this code smell arise when a class does not properly use Object-Oriented programming principles (i.e., inheritance and polymorphism), declares at least one long method with no parameters, and uses instance variables (Brown et al. 1998).
- **Complex Class.** As the name suggests, instances of this smell affect classes that have high values for the Weighted Methods for Class metric (Chidamber and Kemerer 1994)—which is the sum of the cyclomatic complexity (McCabe 1976) of all methods. This smell may primarily make the testing of those classes harder (Fowler and Beck 1999).
- **Inappropriate Intimacy.** This code smell affects classes that use internal fields and methods of another class, hence having a high coupling that might deteriorate program maintainability and comprehensibility (Fowler and Beck 1999).
- **Lazy Class.** The code smell targets classes that do not have enough responsibilities within the system and that, therefore, should be removed to reduce the overall maintainability costs (Fowler and Beck 1999).
- **Refused Bequest.** Classes that only use part of the methods and properties inherited from their parents indicate the presence of possible issues in the hierarchy of the project (Fowler and Beck 1999).
- **Middle Man.** This smell appears when a class mostly delegates its actions to other classes, hence creating a bottleneck for maintainability (Fowler and Beck 1999).

The selected code smells are those more often targeted by related research (Azeem et al. 2019). They have been also connected to an increase of change- and fault-proneness of source code (Catolino et al. 2020; Khomh et al. 2012; Palomba et al. 2018b) as well as maintenance effort (Sjøberg et al. 2012). According to previous work (Khomh et al. 2012; Palomba et al. 2018a; Yamashita and Moonen 2012), all the code smells considered let the affected source code be more prone to changes and faults in different manners. As an example, Palomba et al. (2018a) reported that the change-proneness of classes affected by the *God Class* smell is around 28% higher than classes not affected by the smell, while *Spaghetti Code* increases the change-proneness of classes of about 21%. Other empirical investigations provided different indications, e.g., Khomh et al. (2009) and Khomh et al. (2012) reported that 68% of the classes affected by a *God Class* are also change-prone. As a matter of fact, our current body of knowledge reports that all the code smells we considered are connected to change- and fault-proneness, but different studies provided different estimations on the extent of such connection. In addition, these code smells are highly relevant for developers that, indeed, often recognize them as harmful for the evolvability of software projects (Palomba et al. 2014a; Taibi et al. 2017; Yamashita and Moonen 2013).

#### Selection of Automated Static Analysis Tools

In the context of our research, we selected three well-known automated static analysis tools such as Checkstyle, Findbugs, and PMD. We provide a brief description of these tools in the following:

- **Checkstyle**. Checkstyle is an open-source developer tool that evaluates Java code according to a certain coding standard, which is configured according to a set of “checks”. These checks are classified under 14 different categories, are configured according to the coding standard preference, and are grouped under two severity levels: error and warning. More information regarding the standard checks can be found from the Checkstyle web site.^2^
- **Findbugs**. Findbugs is another commonly used static analysis tool for evaluating Java code, more precisely Java bytecode. The analysis is based on detecting “bug patterns”, which arise for various reasons. Such bugs are classified under 9 different categories, and the severity of the issue is ranked from 1-20. Rank 1-4 is the *scariest* group, rank 5-9 is the *scary* group, rank 10-14 is the *troubling* group, and rank 15-20 is the *concern* group.^3^
- **PMD**. PMD is an open-source tool that provides different standard rule sets for major languages, which can be customized by the users, if necessary. PMD categorizes the rules according to five priority levels (from P1 “Change absolutely required” to P5 “Change highly optional”). Rule priority guidelines for default and custom-made rules can be found in the PMD project documentation.^4^

The selection of these tools was driven by recent findings reporting that these are among the automated static analysis tools more employed in practice by developers (Lenarduzzi et al. 2020; Vassallo et al. 2018; Vassallo et al. 2019). In particular, the most recent of these papers (Vassallo et al. 2019) reported that Checkstyle, PMD, and FindBugs are actually the tools that practitioners use more when developing in Java, along with SonarQube. The selection was therefore based on these observations. In this respect, it is also worth remarking that we originally included SonarQube as well. However, we had to exclude it because it failed on all the projects considered in our study (see Section 3.1.3).

#### Selection of Software Projects

To address the research goals and assess the capabilities of the machine learning techniques for code smell detection, we needed to rely on a dataset reporting actual code smell instances. Most previous studies (Azeem et al. 2019) focused on datasets collected using automated mechanisms, e.g., executing multiple detectors at the same time to consider the instances detected by all of them as actual code smells. Nonetheless, it has been shown that the performance of machine learning-based code smell detectors might be biased by the approximations done, other than by the false positive instances detected when building the ground truth of code smells (Di Nucci et al. 2018b). In this paper, we took advantage of these latter findings and preferred to rely on a manually-labeled dataset containing actual code smell instances. Of course, this choice might have had an impact on the size of the empirical study since there exist only a few datasets of manually-labeled code smells (Azeem et al. 2019). Yet, we were still convinced to opt for this solution, as this was the most appropriate choice to do in order to have reliable results. Indeed, a dataset of real smell instances allowed us to provide reliable results on the performance capabilities of the experimented models and, at the same time, to present a representative case of a real scenario where the code smells arise in similar amounts as in our study (Palomba et al. 2018a) (Table 1).

**Table 1:** Descriptive statistics about the number of code smell instances

Code Smell	Min.	Median	Mean	Max.	Tot.
God Class	0.00	4.00	6.19	23.00	412
Complex Class	0.00	2.00	4.27	16.00	301
Spaghetti Code	0.00	11.00	12.40	32.00	773
Inappropriate Intimacy	0.00	2.00	3.03	10.00	206
Lazy Class	0.00	1.00	1.95	11.00	141
Middle Man	0.00	1.00	1.11	6.00	84
Refused Bequest	0.00	7.00	7.35	17.00	500

From a technical viewpoint, the selection of projects was driven by the above requirement. We exploited a publicly available dataset of code smells developed in previous research (Palomba et al. 2015; Palomba et al. 2018a): this provides a list of 17,350 manually-verified instances of 13 code smell types pertaining to 395 releases of 30 open source systems. Given this initial dataset, we fixed two constraints that the projects to consider had to satisfy. First, the projects had to contain data for the code smells selected in our investigation (see Section 3.1.1). Secondly, we required them to be successfully built so that they could be later analyzed by the selected static analysis tools (see Section 3.1.2). These two constraints were satisfied in 25 releases of the 5 open-source projects reported in Table 2 along their main characteristics.

**Table 2:** Software systems considered in the project

Project	Description	# Classes	# Methods
Apache Ant	Build system	1,218	11,919
Apache Cassandra	Database Management System	727	7,901
Eclipse JDT	Integrated Development Environment	5,736	51,008
HSQLDB	HyperSQL Database Engine	601	11,016
Apache Xerces	XML Parser	542	6,126

For the sake of completeness, it is worth reporting that most of the excluded releases/projects were due to build issues, e.g., dependency resolution problems (Tufano et al. 2017). This possibly remarks the need for additional public code smell datasets composed of projects that can be analyzed through static or dynamic tools.

### Data Collection

The data collection phase aimed at gathering information related to dependent and independent variables of our study. These concern the labeling of code smell instances, namely the identification of real code smells affecting the considered systems, and the collection of static analysis warnings from the selected analyzer, which will represent the features to be used in the machine learners designed in the empirical study.

#### Collecting Information on Actual Code Smell Instances

This stage consisted of identifying real code smells in the considered software projects. The data collection, in this case, was inherited by the dataset exploited. While some previous studies relied on automated mechanisms for this step, e.g., by using metric-based detectors (Arcelli Fontana et al. 2016b; Khomh et al. 2009; Maiga et al. 2012), recent findings showed that such a procedure could threaten the reliability of the dependent variable and, as a consequence, of the entire machine learning model (Di Nucci et al. 2018a). Hence, in our study we preferred a different solution, namely considering manually-validated code smell instances. For all the systems considered, the publicly available dataset exploited in the empirical study report actual code smell instances (Palomba et al. 2015; Palomba et al. 2018a) and has been used in recent studies evaluating the performance of machine learning models for code smell detection (Palomba et al. 2018b; Pecorelli et al. 2019; Pecorelli et al. 2020a). For each code smell, Table 1 reports the distribution of the code smells in the dataset.

#### Collecting Static Analysis Tool Warnings

This step aimed at collecting the data of the independent variables used in our study. Each tool required a different process to collect such data:

- **Checkstyle**. The jar file for the Checkstyle analysis was downloaded directly from the Checkstyle’s website^5^ in order to engage the analysis from the command line. The version of the executable jar file used was the checkstyle-8.30-all.jar. In addition to downloading the jar executable, Checkstyle offers two different types of rule sets for the analysis. For each of the rule sets, the configuration file was downloaded directly from Checkstyle’s guidelines.^6^ In order to start the analysis, the checkstyle-8.30-all.jar and the configuration file in question were saved in the directory where all the projects resided.
- **Findbugs**. FindBugs 3.0.1 was installed by running the brew install findbugs in the command line. Once installed, the GUI was then engaged by writing spotbugs. From the GUI, the analysis was executed through *File* →*NewProject*. The classpath for the analysis was identified to be the location of the project directory. Moreover, the source directories were identified to be the project jar executable. Once the class path and source directories were identified, the analysis was engaged by clicking Analyze in the GUI. Once the analysis finished, the results were saved through *File* →*Saveas* using the XML file format. The main specifications were the ”Classpath for analysis (jar, ear, war, zip, or directory)” and ”Source directories (optional; used when browsing found bugs)” where the project directory and project jar file were added.
- **PMD**. PMD 6.23.0 was downloaded from GitHub^7^ as a zip file. After unzipping, the analysis was engaged by identifying several parameters: project directory, export file format, rule set, and export file name. In addition to downloading the zip file, PMD offers 32 different types of rule sets for Java.^8^ All 32 rule sets were used during the configuration of the analysis.

Using these procedures, we ran the three static analysis tools on the considered software systems. At the end of the analysis, these tools extracted a total of 60,904, 4,707, and 179,020 warnings for Checkstyle, FindBugs, and PMD, respectively.

### Data Analysis

In this section, we report the methodological steps conducted to address our research questions.

#### RQ_1_. Distribution Analysis

To address the first research question, we first showed boxplots depicting the distribution of the metrics and smells. Then, we computed the Mann-Whitney and Cliff’s Delta tests to verify the statistical significance of the observed differences and their effect size. With respect to other possible analyses methods (e.g., correlation), studying the distribution of warnings into the smelly and non-smelly classes not only allowed us to identify the warning types that are more related to code smells, but also to quantify the extent of the difference between the number of warnings contained in smelly and non-smelly classes.

#### RQ_2_ Contribution of Static Analysis Warnings in Code Smell Prediction

In this **RQ**, we assessed the extent to which the various warning categories of the considered static analysis tools can potentially impact the performance of a machine learning-based code smell detector. To this aim, we employed an information gain measure (Quinlan 1986), and particularly the *Gain Ratio Feature Evaluation* technique, to establish a ranking of the features according to their importance for the predictions done by the different models. This analysis method turned to be particularly useful in our case, since it allowed us to precisely quantify the potential predictive power of each warning category for the prediction of code smells. Given a set of features F = {*f*_1_,...,*f*_*n*_} belonging to the model *M*, the *Gain Ratio Feature Evaluation* computes the difference, in terms of Shannon entropy, between the model including the feature *f*_*i*_ and the model that does not include *f*_*i*_ as independent variable. The higher the difference obtained by a feature *f*_*i*_, the higher its value for the model. The outcome is represented by a ranked list, where the features providing the highest gain are put at the top. This ranking was used to address **RQ**_2_.

#### RQ_3_. The Role of Static Analysis Warnings in Code Smell Prediction

Once we had investigated which warning categories relate the most to the presence of code smells, in **RQ**_3_ we proceeded with the definition of machine learning models. Specifically, we defined a feature for each warning type raised by the tools, where each feature contained the number of violations of that type identified in a class. For instance, suppose that for a class C_*i*_ Checkstyle identifies seven violations to the warning type called *“Bad Practices”*: the machine learner is fed with the integer value “7” for the feature *“Bad Practices”* computed on the class C_*i*_.

The dependent variable was, instead, given by the presence/absence of a certain code smell. This implied the construction of seven models for each tool, i.e., for each static analysis tool considered, we built a model that used its warnings types as features to predict the presence of *God Class*, *Spaghetti Code*, *Complex Class*, *Inappropriate Intimacy*, *Lazy Class*, *Refused Bequest*, and *Middle Man*. Overall, this design led to the creation of 21 models per project, i.e., one for each code smell/static analysis tool pair. For the sake of clarity, it is worth remarking that we considered each release of the projects in the dataset as an independent project. This choice was taken after an in-depth investigation of the differences among the releases available: we indeed discovered that the releases that met our filtering criteria (see Section 3.1.3) were too far in time from each other, making other strategies unfeasible/unreliable—as an example, the excessive distance among releases made not feasible a release-by-release methodology where subsequent releases are considered following a time-sensitive data analysis (Pascarella et al. 2019; Tantithamthavorn and Hassan 2018).

As for the supervised learning algorithm, the literature in the field still misses a comprehensive analysis of which algorithm works better in the context of code smell detection (Azeem et al. 2019). For this reason, we experimented with multiple classifiers such as *J48*, *Random Forest*, *Naive Bayes*, *Support Vector Machine*, and *JRip*. When training these algorithms, we followed the recommendations provided by previous research (Azeem et al. 2019; Tantithamthavorn and Hassan 2018) to define a pipeline dealing with some common issues in machine learning modeling. In particular, we exploited the output of the Gain Information algorithm—used in the context of **RQ**_2_—to discard irrelevant features that could bias the interpretation of the models (Tantithamthavorn and Hassan 2018): we did that by excluding the features not providing any information gain. We also configured the hyper-parameters of the considered machine learners using the MultiSearch algorithm (Ye and Kalyanaraman 2003), which implements a multidimensional search of the hyper-parameter space to identify the best configuration of the model based on the input data. Finally, we considered the problem of data balancing: it has been recently explored in the context of code smell prediction (Pecorelli et al. 2020a) and the reported findings showed that data balancing may or may not be useful to improve the performance of a model. Hence, before deciding on whether to apply data balancing, we benchmarked (i) *Class Balancer*, which is an oversampling approach (ii) *Resample*, an undersampling method (iii) *Smote*, an approach including synthetic instances to oversample the minority class, and (iv) *NoBalance*, namely the application of no balancing methods.

After training the models, we proceeded with the evaluation of their performance. We applied a 10-fold cross-validation, as it allows to verify multiple times the performance of a machine learning model built using various training data against unseen data. With this strategy, the dataset (including the training set) was divided in 10 parts respecting the proportion between smelly and non-smelly elements. Then, we trained for ten times the models using 9/10 of the data, retaining the remaining fold for testing purpose—in this way, we allowed each fold to be the test set exactly once. For each test fold, we evaluated the models by computing a number of performance metrics, such as precision, recall, F-Measure, AUC-ROC, and Matthews Correlation Coefficient (MCC). Finally, with the aim of drawing statistically significant conclusions, we applied the post-hoc Nemenyi test (Nemenyi 1962) on the distributions of MCC values achieved by the experimented machine learners, setting the significance level to 0.05.

#### RQ_4_. Orthogonality Between the Three Single-Tool Prediction Models

When addressing this research question, we were interested in understanding whether the different machine learners experimented in the context of **RQ**_3_ were able to detect code smell instances that are not detected also by other techniques. If this was the case, then it meant that different automated static analysis tools would have had the potential to predict the smelliness of classes differently, hence possibly enabling the definition of a combined machine learning mechanism that it could have further improved the code smell detection capabilities. In other terms, the analysis aimed at understanding how many true positives can be identified by a specific model alone and how many true positives can be correctly identified by multiple models. To this purpose, for each code smell type, we compared the sets of *correctly* detected instances by a technique *m*_*i*_ with those identified by an alternative technique *m*_*j*_ using the following overlap metrics (Oliveto et al. 2010):

$$ correct_{m_{i} \cap m_{j}} = {|correct_{m_{i}} \cap correct_{m_{j}}| \over |correct_{m_{i}} \cup correct_{m_{j}}|} \% $$

$$ correct_{m_{i} \setminus m_{j}} = {|correct_{m_{i}} \setminus correct_{m_{j}}| \over |correct_{m_{i}} \cup correct_{m_{j}}|} \% $$

where $correct_{m_{i}}$ represents the set of correct code smells detected by the approach *m*_*i*_, $correct_{m_{i} \cap m_{j}}$ measures the overlap between the set of true code smells detected by both approaches *m*_*i*_ and *m*_*j*_, and $correct_{m_{i} \setminus m_{j}}$ appraises the true smells detected by *m*_*i*_ only and missed by *m*_*j*_. The latter metric provides an indication of how a code smell detection technique contributes to enriching the set of correct code smells identified by another approach.

We also considered an additional orthogonality metric, which computed the percentage of code smell instances correctly identified only by the prediction model *m*_*i*_. In this way, we could measure the extent to which the warning types of a specific static analysis tool contributed to the identification of all correct instances identified. Specifically, we computed:

$$ correct_{m_{i} \setminus (m_{j} \cup m_{k})} = {|correct_{m_{i}} \setminus (correct_{m_{j}} \cup correct_{m_{k}})| \over |correct_{m_{i}} \cup correct_{m_{j}} \cup correct_{m_{k}}|} \% $$

While different models can identify different correct code smell instances, they can also identify different false positives. This means that the complementarity of the models does not necessarily mean that their combination would result in a better model. In the next Section we show how to build a combined model and compare it with the individual ones.

#### RQ_5_. Toward a Combination of Automated Static Analysis Tools for Code Smell Prediction

In this research question, we took into account the possibility to devise a combined model that mixes together the outputs of different static analysis tools.

Starting from all warning types of the various tools, we have proceeded as follows. In the first place, we built a new dataset where, for all classes of the systems considered, we reported all the warnings raised by all tools. This step led to the creation of unique dataset that combined all the information mined in the context of our previous research questions. In the second place, we have re-run the *Gain Ratio Feature Evaluation* (Quinlan 1986) in order to globally rank the features and discard those that, in such a new combined dataset, did not provide any information gain.

After discarding the irrelevant features, we have followed the same steps as **RQ**_3_ with the aim of conducting a fair comparison of the combined model with the individual ones previously experimented. As such, we trained the model using multiple classifiers appropriately configured using the MultiSearch algorithm (Ye and Kalyanaraman 2003) and considering the problem of data balancing (Pecorelli et al. 2020a). Afterwards, to verify the performance of the combined model, we adopted the same validation strategy as **RQ**_3_ and compared it with the values of F-Measure, AUC-ROC, and Matthews Correlation Coefficient obtained by the individual models. Finally, we used the Nemenyi test (Nemenyi 1962) for statistical significance.

#### RQ_6_. Comparison with a Baseline Machine Learner

To address **RQ**_6_, we had to first select an existing solution to compare with. Most of the previous studies (Al-Shaaby et al. 2020; Azeem et al. 2019; Kaur et al. 2021) experimented with various machine learning techniques, yet they all employed code metrics as predictors. As an example, Maiga et al. (2012b) characterized *God Class* instances by means of Object-Oriented metrics. Similarly, other researchers have attempted to verify how different machine learning algorithms work in the task of code smell classification without focusing on the specific features to use for this purpose (Azeem et al. 2019). Hence, we decided to devise a baseline machine learning technique that uses code metrics as predictors. In this respect, we computed the entire set of metrics proposed by Chidamber and Kemerer’s suite (Chidamber and Kemerer 1994) with our own tool and use them as features.

After computing the code metrics, we followed exactly the same methodological procedure used in the context of **RQ**_3_ and **RQ**_5_. As such, the baseline machine learner aimed at predicting the presence/absence of code smells. Also in this case, we experimented with various machine learning algorithms, finding *Random Forest* to be the best one. When training the baseline, we took care of possible multi-collinearity by excluding the code metrics providing no information gain, other than tuning the hyper-parameters by means of the MultiSearch algorithm (Ye and Kalyanaraman 2003). In terms of data balancing, we verified what was the best possible configuration, benchmarking *Class Balancer*, *Resample*, *Smote*, and *NoBalance*: *Smote* was found to be the best option.

We applied a 10-fold cross validation on the dataset, so that we could have a fair comparison with the approach devised in **RQ**_5_—note that we did not consider a full comparison with the individual models experimented in **RQ**_3_ since these were shown already to be less performing. The accuracy of the baseline was assessed through F-Measure, AUC-ROC, and MCC. Finally, we executed the post-hoc Nemenyi test (Nemenyi 1962) on the distributions of MCC values achieved by the baseline and the combined machine learner output by **RQ**_5_, setting the significance level to 0.05.

#### RQ_7_. Orthogonality Between the Warning- and Metric-Based Prediction Models

In this research question we performed a complementarity analysis between the warning- and the metric-based Prediction Models. In order to perform such a complementarity analysis, we followed the same methodology applyed for **RQ**_4_. In particular, for each actual smelly instance, we computed the overlap metrics described in Section 3.3.4, i.e., $correct_{m_{i} \cap m_{j}}$ and $correct_{m_{i} \setminus m_{j}}$.

#### RQ_8_. Combining Static Analysis Warnings and Code Metrics

To study the performance of a machine learner that exploits both static analysis warnings and code metrics, we have proceeded in a similar manner as the other research questions, After combining all the metrics experimented so far in a unique dataset, we re-run the *Gain Ratio Feature Evaluation* (Quinlan 1986) to understand the contribution provided by each of those metrics. As previously done, we discarded the ones whose contribution was null. Afterwards, we followed the same steps as **RQ**_5_ and compared the performance of the combined model to the previously built models using F-Measure, AUC-ROC, and MCC, other than the Nemenyi test (Nemenyi 1962) for statistical significance.

## Analysis of the Results

In the following, we discuss the results achieved when addressing our research questions. For the sake of understandability, we report the discussion by RQ.

### RQ_1_. Distribution Analysis

**Fig. 1:**
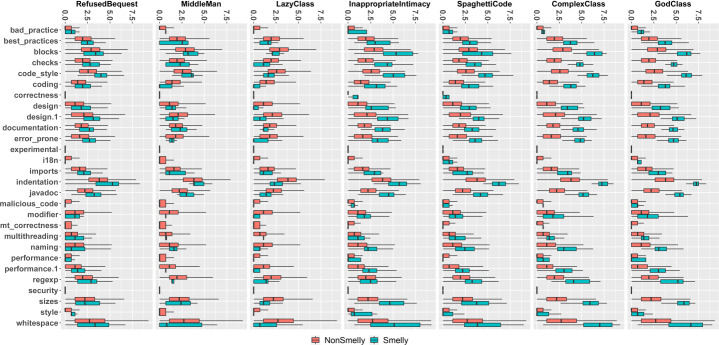
Boxplots reporting warnings distributions in smelly/non smelly classes for the seven code smells considered

Figure 1 shows boxplots of the distributions of warning categories in smelly and non-smelly classes for the seven code smell types considered in the study. Regardless of the code smell and the warning category considered, the distributions always contain higher values for smelly cases, i.e., smelly classes are more likely to contain a higher number of warnings. The only exception is represented by *Lazy Class*, in which the greater number of warnings arises in classes that are not affected by this code smell. Although this result could sound strange, it is fair to remember that *Lazy Class* refers to very short classes that basically have no responsibility. Therefore, it is reasonable to think that lazy classes are associated with few or no warnings. Table 3 reports results for the Mann-Whitney and Cliff’s Delta tests. Results indicate that for most of the warning categories, there is a statistically significant difference between the two distributions, thus indicating that those categories represent relevant features to discriminate smelly and non-smelly instances. Turning to the analysis of the categories related to each individual tool, we can see that *PMD* yields the most relevant warnings. Indeed, except for *Middle Man* and *Lazy Class*, all the warning categories belonging to this tool resulted to be relevant. Similarly, *Checkstyle*’s warning categories are very relevant for six out of the seven code smells considered. Finally, the warnings generated by *Findbugs* are those showing the smaller differences between the two considered distributions. 
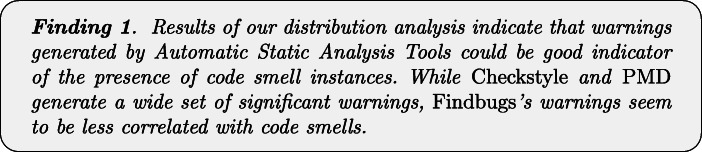

**Table 3:** Mann Whitney and Cliff’s Delta Statistical Test Results

		God Class		Complex Class		Spaghetti Code		Inapp. Intimacy		Lazy Class		Middle Man		Refused Bequest	
Tool	Warning	p-value	*δ*	p-value	*δ*	p-value	*δ*	p-value	*δ*	p-value	*δ*	p-value	*δ*	p-value	*δ*
Checkstyle	regexp	**3.2e-68**	**M**	**9.9e-66**	**M**	4.1e-02	N	3.1e-04	N	2.5e-01	N	**8.7e-08**	**S**	**9.9e-06**	**N**
	checks	**1.6e-86**	**L**	**1.7e-57**	**L**	**3.3e-13**	**N**	**4.2e-23**	**M**	**1.8e-08**	**S**	**1.7e-04**	**S**	**1e-15**	**S**
	whitespace	**3e-93**	**L**	**1.6e-69**	**L**	**2.6e-17**	**S**	**1e-25**	**M**	8.5e-01	N	**4.6e-05**	**S**	**1.1e-15**	**S**
	blocks	**1.5e-100**	**L**	**3.8e-68**	**L**	**1.2e-20**	**S**	**1.6e-36**	**M**	7.7e-01	N	**3.3e-18**	**L**	**1.2e-18**	**S**
	sizes	**3.2e-77**	**L**	**9.7e-50**	**L**	**1.7e-04**	**N**	**4.9e-23**	**M**	8.7e-01	N	7.4e-01	N	6.4e-02	N
	javadoc	**2.2e-74**	**L**	**3.8e-46**	**L**	**1.4e-10**	**N**	**3.8e-23**	**M**	7e-04	S	**1e-09**	**M**	**2.2e-10**	**S**
	indentation	**3.1e-60**	**M**	**1e-38**	**M**	**1.1e-12**	**N**	**2.6e-15**	**S**	5.2e-03	N	1.7e-04	S	2.1e-04	N
	naming	**1.4e-128**	**L**	**2.8e-78**	**L**	**4.8e-39**	**S**	**2.3e-29**	**M**	3.7e-02	N	9.9e-01	N	**2.8e-11**	**N**
	imports	**1.1e-40**	**M**	**5.7e-27**	**M**	3.3e-02	N	**4.2e-22**	**M**	7.5e-02	N	5.8e-01	N	**4.6e-06**	**N**
	coding	**2.2e-114**	**L**	**2.3e-77**	**L**	**2e-43**	**S**	**1.2e-35**	**M**	1.7e-01	N	1.8e-01	N	**5.8e-08**	**N**
	design	**1.2e-68**	**M**	**1.5e-39**	**M**	**2.5e-11**	**N**	**1e-23**	**M**	3.8e-03	N	**5.8e-12**	**M**	**3.4e-05**	**N**
	modifier	**6e-136**	**M**	**4.9e-103**	**M**	**1.9e-17**	**N**	**1.3e-47**	**S**	8.1e-01	N	3.4e-01	N	1.5e-01	N
Findbugs	style	**1.1e-63**	**S**	**7.9e-20**	**N**	**2.2e-120**	**S**	**4.2e-19**	**N**	4.9e-01	N	7.3e-02	N	**9.2e-07**	**N**
	correctness	**2e-07**	**N**	1.7e-02	N	**4.1e-25**	**N**	4.7e-02	N	6.1e-01	N	5.6e-01	N	1.3e-01	N
	performance	**1.2e-13**	**N**	**2.5e-19**	**N**	**2.5e-23**	**N**	**1.5e-37**	**N**	9.6e-01	N	2.8e-01	N	**8.2e-07**	**N**
	malicious_code	1.1e-04	N	1.3e-01	N	1.2e-04	N	**8.8e-12**	**N**	5.2e-01	N	3.1e-01	N	4.2e-01	N
	bad_practice	**7.3e-23**	**N**	5.6e-03	N	**2.5e-112**	**N**	**2.4e-36**	**S**	1.3e-01	N	**3.4e-08**	**N**	8.5e-03	N
	i18n	**3.5e-10**	**N**	4e-03	N	**4e-101**	**N**	**8.3e-08**	**N**	4.1e-01	N	2.6e-01	N	1.8e-01	N
	mt_correctness	**2.1e-10**	**N**	3e-01	N	**2.9e-21**	**N**	**4.4e-26**	**N**	5e-01	N	6.1e-01	N	1.9e-01	N
	experimental	5.5e-01	N	6.2e-01	N	**6.4e-18**	**N**	6.6e-01	N	7.4e-01	N	8e-01	N	5.2e-01	N
	security	7.7e-01	N	8.1e-01	N	**1.1e-79**	**N**	8.3e-01	N	8.7e-01	N	9e-01	N	7.5e-01	N
PMD	documentation	**4.1e-233**	**L**	**2.9e-145**	**L**	**1.9e-190**	**L**	**7.7e-70**	**L**	**2.9e-09**	**S**	3.2e-03	S	**4.6e-31**	**S**
	code_style	**6.5e-233**	**L**	**2e-160**	**L**	**1.5e-302**	**L**	**8.3e-73**	**L**	**1.3e-08**	**S**	**2.8e-05**	**S**	**3.3e-79**	**L**
	best_practices	**3.6e-166**	**L**	**3.1e-120**	**L**	**1.3e-210**	**L**	**2e-43**	**L**	9.9e-03	N	8.9e-01	N	**1.2e-66**	**M**
	design	**1.6e-236**	**L**	**1.1e-164**	**L**	**0e + 00**	**L**	**1.8e-62**	**L**	**1.3e-06**	**S**	7.4e-01	N	**2e-63**	**M**
	error_prone	**4.2e-239**	**L**	**1.9e-162**	**L**	**0e + 00**	**L**	**2.1e-59**	**L**	1.3e-04	S	1.7e-01	N	**3.9e-67**	**M**
	multithreading	**3.7e-177**	**M**	**5.3e-109**	**M**	**4.2e-93**	**S**	**1.3e-22**	**S**	8.9e-01	N	3.6e-01	N	**1.3e-16**	**N**
	performance	**1.2e-285**	**L**	**4.7e-204**	**L**	**0e + 00**	**L**	**2.2e-95**	**L**	**5.3e-08**	**S**	6.8e-01	N	**7.5e-62**	**M**

We use N, S, M, and L to indicate negligible, small, medium and large effect size respectively. Significant p-values and *δ* values are reported in bold-face

### RQ_2_. Contribution of static analysis warnings in code smell prediction.

Table 4 reports the mean information gain values obtained by the metrics composing the 21 models built in our study. For the sake of readability, we just reported the three most relevant warning categories for each model, i.e., one for each tool-smell combination—the interested reader can find the complete results as part of our online appendix (Pecorelli et al. 2021).

**Table 4:** Information Gain of our independent variables for each static analysis tool

	Checkstyle		FindBugs		PMD	
Code Smell	Metric	Mean	Metric	Mean	Metric	Mean
God Class	Indentation	0.03	Style	0.02	Code Style	0.03
	Blocks	0.03	Bad Practice	0.01	Documentation	0.03
	Sizes	0.03	I18N	0.01	Error Prone	0.03
Complex Class	Indentation	0.04	Style	0.02	Code Style	0.03
	Blocks	0.04	Security	0.01	Design	0.03
	Sizes	0.03	Malicious Code	0.01	Error Prone	0.03
Spaghetti Code	Indentation	0.03	I18N	0.01	Error Prone	0.03
	Blocks	0.02	Security	0.01	Code Style	0.03
	Coding	0.02	Correctness	0.01	Design	0.03
Inappropriate Intimacy	Whitespace	0.01	Bad Practice	0.02	Code Style	0.01
	Indentation	0.01	Style	0.01	Error Prone	0.01
	Javadoc	0.01	Correctness	0.01	Design	0.01
Lazy Class	Javadoc	0.01	Security	0.01	Code Style	0.01
	Sizes	0.01	Malicious Code	0.01	Documentation	0.01
	Indentation	0.01	Correctness	0.01	Design	0.01
Middle Man	Indentation	0.01	Security	0.01	Error Prone	0.01
	Design	0.01	Malicious Code	0.01	Documentation	0.01
	Checks	0.01	Correctness	0.01	Code Style	0.01
Refused Bequest	Indentation	0.01	Style	0.01	Code Style	0.01
	Checks	0.01	Security	0.01	Error Prone	0.01
	Design	0.01	Malicious Code	0.01	Design	0.01

Looking at the achieved results, the first thing to notice is that, depending on the code smell type, the warning types could have different weights: this practically means that a machine learner for code smell identification should exploit different features depending on the target code smell rather than rely on a unique set of metrics to detect them all. As an example, the *Indentation* type of Checkstyle provides different information gain based on the specific code smell type. This seems to suggest that not all warnings would have the same impact on the performance of various code smell detectors.

When analyzing the most powerful features of Checkstyle and PMD, we could notice that features related to source code readability are constantly at the top of the ranked list for all the considered code smells. This is, for instance, the case of the *Indentation* warnings given by Checkstyle or the *Code Style* metrics highlighted by PMD. The most relevant warnings also seem to be strongly related to specific code smells: as an example, the presence of a high number of blocks having a large size might strongly affect the likelihood to have a *God Class* or or a *Complex Class* smell; similarly, design-related issues are the most characterizing aspects of a *Spaghetti Code* or a *Middle Man*. In other words, from this analysis, we could delineate a relation between the most relevant warnings highlighted by Checkstyle and PMD and the specific code smells considered in this paper.

A different discussion should be done for FindBugs: in this case, the most powerful metrics mostly relate to *Performance* or *Security*, which are supposed to cover different code issues than code smells. As such, we expect this static analysis tool to have lower performance when used for code smell detection.

Finally, it is worth noting that the information gain of the considered features seems to be generally low. On the one hand, this may potentially imply a low capability of the features when employed within a machine learning model. On the other hand, it may also be the case that such a little information would already be enough to characterize and predict the existence of code smell instances. The next sections address this point further (Table 5). 
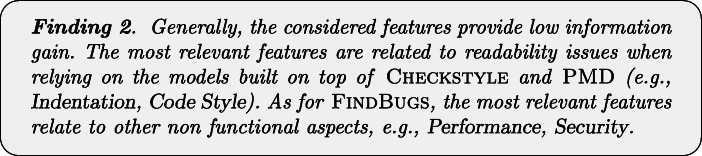

**Table 5:** Aggregate results reporting the performance of the models built with the warning generated by the three static automatic tools

	Checkstyle				FindBugs				PMD			
	Prec.	Recall	FM	MCC	Prec.	Recall	FM	MCC	Prec.	Recall	FM	MCC
God Class	0.01	0.62	0.02	0.04	0.01	0.25	0.01	0.01	0.43	0.52	0.47	0.47
Complex Class	0.01	0.48	0.01	0.02	0.00	0.22	0.01	0.00	0.28	0.35	0.31	0.31
Spaghetti Code	0.02	0.43	0.03	0.05	0.01	0.19	0.02	0.00	0.26	0.22	0.24	0.23
Inappropriate Intimacy	0.01	0.44	0.01	0.03	0.00	0.31	0.00	-0.01	0.08	0.17	0.11	0.11
Lazy Class	0.01	0.13	0.01	0.02	0.00	0.63	0.00	-0.01	0.04	0.11	0.06	0.06
Middle Man	0.00	0.15	0.00	-0.02	0.00	0.66	0.00	0.01	0.08	0.03	0.04	0.05
Refused Bequest	0.01	0.38	0.01	0.00	0.01	0.50	0.01	0.00	0.27	0.14	0.18	0.19

### RQ_3_. The role of static analysis warnings in code smell prediction.

**Fig. 2:**
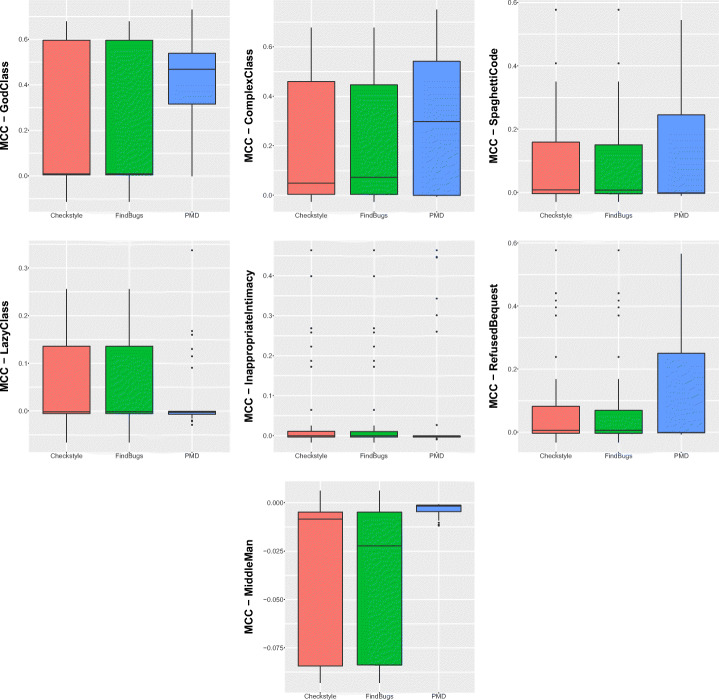
Boxplots representing the MCC values obtained by Random Forest trained on static analysis warnings for code smells detection

Figure 2 reports the performance capabilities in terms of MCC of the models built using the warnings given by Checkstyle, FindBugs, and PMD, respectively. In this section, we only discuss the overall results obtained with the best configuration of the models, namely the one considering *Random Forest* as classifier and *Class Balancer* as data balancing algorithm. The results for the other models are available in our online appendix (Pecorelli et al. 2021).

We can immediately point out that the models built using the warnings of static analysis tools have very low performance. In almost all cases, indeed, the MCCs show median values that are very close to zero, indicating a very low, if not even null correlation between the set of detected and the set of actual smelly instances. This result is in line with previous studies on the application of machine learning for code smell detection (Di Nucci et al. 2018b; Pecorelli et al. 2019). As an example, Pecorelli et al. (2019) reported that models built using code metrics of the Chidamber-Kemerer suite (Chidamber and Kemerer 1994) work worst than a constant classifier that always considers an instance as non-smelly. Perhaps more interestingly, our findings contradict the preliminary insights we obtained on the capabilities of static analysis warnings as features for code smell detection (Lujan et al. 2020a): indeed, when replicating the study on a larger scale, we could not confirm the fairly high performance previously achieved, highlighting how replications in software engineering research represent a precious method to corroborate (or not) analyses done under specific conditions that can affect generalizability (Carver et al. 2014).

The reasons behind the low MCC values could be various. This coefficient is computed by combining true positives, true negatives, false positives, and false negatives altogether; as such, having a clear understanding of the factors impacting those values is not trivial. In an effort of determining these reasons, Table 4 provides a more detailed overview of the performance of the models for each of the considered tools and code smells.

The first aspect to consider is that, when considering Checkstyle and FindBugs, the low performance could be due to the high false-positive rate. Indeed, despite the moderately high recall, the results are negatively influenced by the very low precision that is always close to zero. A different conclusion must be drawn for PMD. The results show similar precision and recall values when considering the code smells individually, but these values are higher or lower depending on the specific code smell type. In other words, our results indicate that the models built using the warnings provided by this tool could achieve higher or lower performance, depending on the smell considered—hence, the capabilities of these models cannot be generalized to all code smells.

Another important aspect to take into account is the different behaviour of the three models with respect to the code smell to detect. While Checkstyle and PMD achieve better performance in detecting *God Class*, *Complex Class*, and *Spaghetti Code*, FindBugs gives its best in the detection of *Lazy Class*, *Middle Man*, and *Refused Bequest*.

**Fig. 3:**
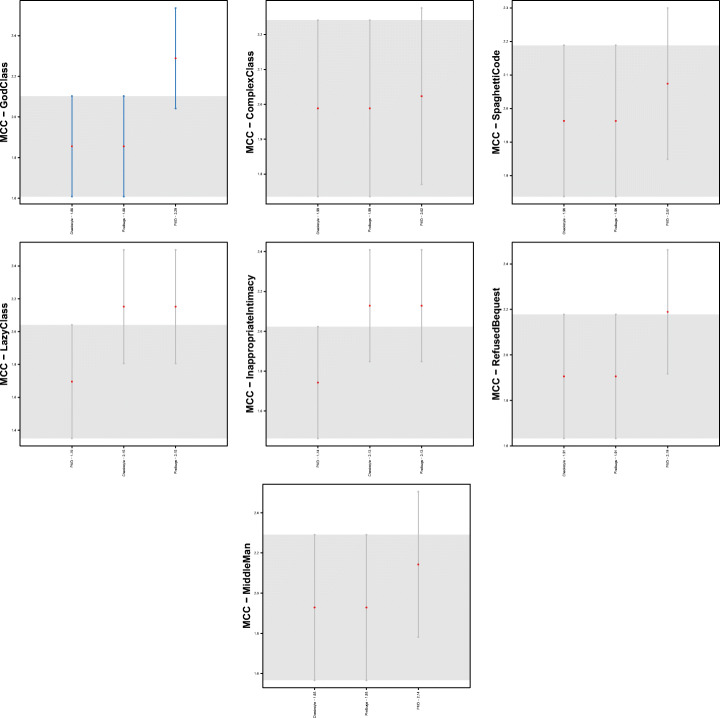
Plots representing the results of Nemenyi test for statistical significance between the MCC values obtained by Random Forest trained on static analysis warnings for code smells detection

Figure 3 confirms the discussion above. Indeed, by analyzing the statistical difference between models with respect to code smells, we can notice that PMD performance are statistically better than the other two models when detecting *God Class* instances. In the cases of *Lazy Class* and *Inappropriate Intimacy* code smells, instead, models built with warning generated by Checkstyle, and FindBugs performs significantly better than those relying on PMD warnings.

Nonetheless, despite the negative results achieved so far, it is worth reflecting on two specific aspects coming from our analysis. On the one hand, for each code smell there is at least one tool whose warnings are able to catch a good number of smelly instances (i.e., recall ≈ 50%). On the other hand, different warning categories achieve higher performance on different sets of code smells. Based on these two considerations, we conjectured that higher performance could be potentially achieved when combining the warnings generated by the three static analysis tools. Next paragraphs address this point deeply. 
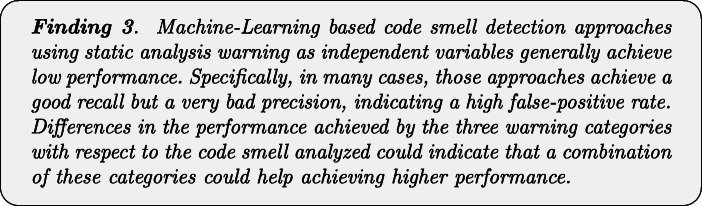


### RQ_4_. Orthogonality of the Prediction Models

In the context of the fourth research question, we sought to move toward a combination of warning types coming from different static analysis tools for code smell detection. Let discuss the results by analyzing Table 6, that reports the overlap between the model using the warnings generated by Checkstyle and the one built on the FindBugs warnings. It is interesting to observe that there is a very high complementarity between the two models, regardless on the code smell considered. Indeed, only a small portion of smelly instances are correctly identified by both the models, i.e., (*CS* ∩ *FB*) ≤ 21%. Moreover, the percentage of instances correctly classified by only one of the models is generally high, indicating such complementarity.

**Table 6:** Overlap analysis between Checkstyle and Findbugs

Code Smell	CS ∩ FB	CS ∖ FB	FB ∖ CS
God Class	7%	47%	46%
Complex Class	11%	37%	52%
Spaghetti Code	5%	70%	25%
Inappropriate Intimacy	8%	23%	69%
Lazy Class	0%	7%	93%
Middle Man	8%	0%	92%
Refused Bequest	21%	25%	54%

Table 7 show the results of the overlap between the models built on Checkstyle and PMD warnings. The table immediately suggests that PMD provides a very limited contribution in terms of new smelly instances discovered. Results suggest that for all code smells, Checkstyle alone could detect almost the same set of smelly instances.

**Table 7:** Overlap analysis between Checkstyle and PMD

Code Smell	CS ∩ PMD	CS ∖ PMD	PMD ∖ CS
God Class	0%	98%	2%
Complex Class	0%	98%	2%
Spaghetti Code	2%	94%	4%
Inappropriate Intimacy	33%	60%	7%
Lazy Class	0%	100%	0%
Middle Man	0%	100%	0%
Refused Bequest	0%	100%	0%

Table 8 provides the overlap results for FindBugs and PMD. These results deserve a discussion similar to the previous one. Indeed, as we discussed above, also in this case PMD does not provide an important contribution. Most of the correctly classified instances are indeed provided by the model built only on FindBugs warnings.

**Table 8:** Overlap analysis between Findbugs and PMD

Code Smell	FB ∩ PMD	FB ∖ PMD	PMD ∖ FB
God Class	1%	98%	1%
Complex Class	0%	98%	2%
Spaghetti Code	2%	87%	11%
Inappropriate Intimacy	10%	84%	6%
Lazy Class	0%	100%	0%
Middle Man	0%	100%	0%
Refused Bequest	0%	100%	0%

Finally, looking at the overlap results for all the three models, shown in Table 9, we can confirm the above results. The low percentage of instances that are simultaneously correctly detected as smelly by all three approaches indicates a high complementarity between the instances detected by the three tools, i.e., different tools are able to detect different sets of smelly instances. Such complementarity is an indicator that better performance could be achieved by combining the warnings generated by the three tools in a unique, unified, detection model. 
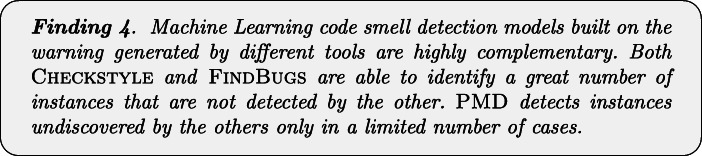

**Table 9:** Overlap Analysis considering each tool independently

Code Smell	CS ∖ (FB ∪ PMD)	FB ∖ (CS ∪ PMD)	PMD ∖ (CS ∪ FB)	CS ∩ FB ∩ PMD
God Class	44%	56%	0%	0%
Complex Class	38%	59%	2%	0%
Spaghetti Code	74%	23%	2%	1%
Inappropriate Intimacy	40%	46%	1%	13%
Lazy Class	4%	95%	1%	0%
Middle Man	21%	79%	0%	0%
Refused Bequest	36%	62%	2%	0%

### RQ_5_. Toward a Combination of Automated Static Analysis Tools for Code Smell Prediction

In the context of this **RQ**, we defined and evaluated a combined model. As explained in Section 4.2, we faced the problem by first measuring the potential information gain by the warning types when put all together and then considering the most relevant warnings for the definition of a more effective combination. Table 10 reports the information gain values obtained by the metrics composing the combined models. Also in this case, for the sake of readability we only reported the three most relevant categories for each model. The complete results can be found in our online appendix (Pecorelli et al. 2021).

**Table 10:** Information Gain of our independent variables for the combined model

	Combined model	
Code Smell	Metric	Mean
God Class	Code.Style	0.03
	Documentation	0.02
	Design	0.02
Complex Class	Code Style	0.03
	Design	0.02
	Error Prone	0.02
Spaghetti Code	Error Prone	0.03
	Code Style	0.02
	Design	0.02
Inappropriate Intimacy	Code Style	0.01
	Whitespace	0.01
	Design	0.01
Lazy Class	Javadoc	0.01
	Sizes	0.01
	Code Style	0.01
Middle Man	Imports	0.01
	Design	0.01
	Checks	0.01
Refused Bequest	Code Style	0.01
	Error Prone	0.01
	Documentation	0.01

Looking at the table, the first consideration we can do is that readability-related features remain relevant even when considering all the features together. Some examples are *Code Style* for *God Class* or *Javadoc* for *Lazy Class*. Differently, features related to performance and security aspects, that have been shown to be relevant in the models built only on FindBugs warnings, are no longer important when combining the tools.

Another important aspect is related to the presence of design-related features in the list of the most relevant predictors. Those features, that are the more in-line with the definition of code smell, were surprisingly excluded in the context of our **RQ**_2_. The fact that they become more relevant when the three tools are combined may represent an indicator of the fact that a combined model can outperform the models discussed in **RQ**_3_.

**Fig. 4:**
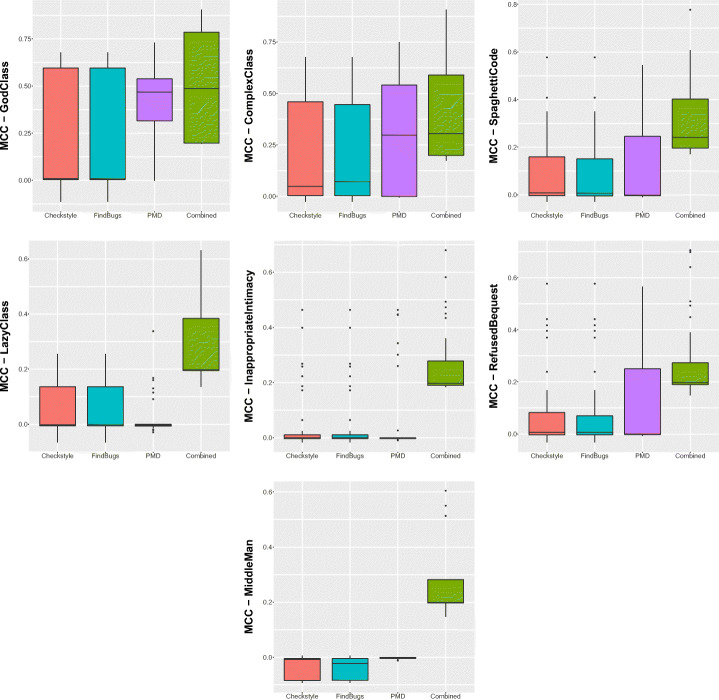
Boxplots representing the MCC values obtained by Random Forest trained on static analysis warnings for code smells detection

**Fig. 5:**
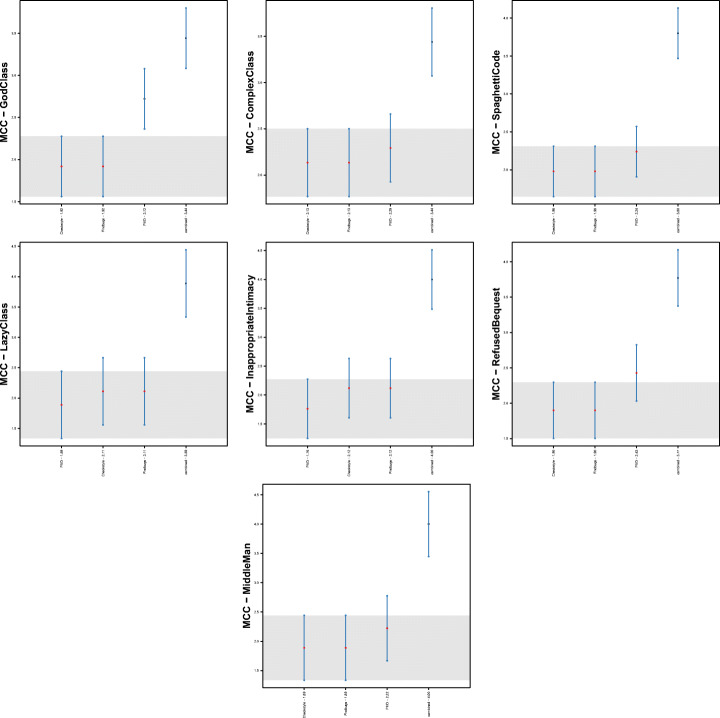
Plots representing the results of Nemenyi test for statistical significance between the MCC values obtained by Random Forest trained on static analysis warnings for code smells detection

Table 11 and Fig. 4 show the performance of the combined model. As we can see, there is a general improvement, particularly in terms of precision—hence confirming our hypothesis on the potential of combining features of different static analysis tools to reduce false positives. The MCC values, ranging between 14% and 48% are clearly better than the one provided by the single models, as discussed in **RQ**_3_. Results of Nemenyi test, reported in Fig. 5, evidenced a clear statistical difference between the MCCs achieved by the combined model and the ones provided by single-tool models. However, unfortunately, these results still indicate the unsuitability of machine learning approaches for code smell detection, as already proven in previous studies in the field (Di Nucci et al. 2018b; Pecorelli et al. 2019). A more detailed discussion of what these findings imply for code smell research and, particularly, for the applicability of machine learning solutions to detect code smells is reported in Section 5. 
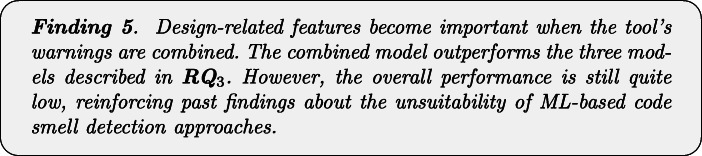

**Table 11:** Results reporting the performance of the model built by combining the warning generated by the three static automatic tools

	Checkstyle				FindBugs				PMD				Combined			
	Prec.	Recall	FM	MCC	Prec.	Recall	FM	MCC	Prec.	Recall	FM	MCC	Prec.	Recall	FM	MCC
God Class	0.01	0.62	0.02	0.04	0.01	0.25	0.01	0.01	0.43	0.52	0.47	0.47	0.49	0.47	0.48	0.48
Complex Class	0.01	0.48	0.01	0.02	0.00	0.22	0.01	0.00	0.28	0.35	0.31	0.31	0.34	0.34	0.34	0.34
Spaghetti Code	0.02	0.43	0.03	0.05	0.01	0.19	0.02	0.00	0.26	0.22	0.24	0.23	0.31	0.19	0.24	0.24
Inappropriate Intimacy	0.01	0.44	0.01	0.03	0.00	0.31	0.00	-0.01	0.08	0.17	0.11	0.11	0.21	0.15	0.17	0.17
Lazy Class	0.01	0.13	0.01	0.02	0.00	0.63	0.00	-0.01	0.04	0.11	0.06	0.06	0.17	0.12	0.14	0.14
Middle Man	0.00	0.15	0.00	-0.02	0.00	0.66	0.00	0.01	0.08	0.03	0.04	0.05	0.56	0.07	0.13	0.20
Refused Bequest	0.01	0.38	0.01	0.00	0.01	0.50	0.01	0.00	0.27	0.14	0.18	0.19	0.39	0.09	0.15	0.18

### RQ_6_. Comparison with a Baseline Machine Learner

**Fig. 6:**
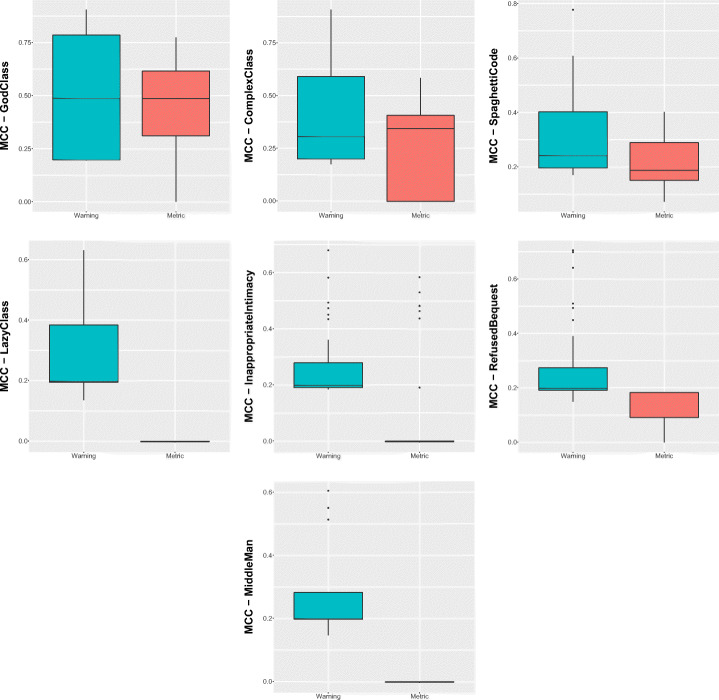
Boxplots representing the MCC values obtained by Random Forest trained on static analysis warnings and structural metrics for code smells detection

Table 12 and Fig. 6 report the results regarding the comparison of the performance achieved by the model that uses the combination of the warnings generated by the three ASATs considered, and the model using structural information as predictors. The first consideration is that the model using the warnings generated by the three ASATs seems to slightly outperform the model using structural information for almost all the code smell types. In particular, this is the case of *Lazy Class*, *Inappropriate Intimacy*, *Refused Bequest*, and *Middle Man*. These four smells do not have a direct correlation with structural information given to the structural classifier. For instance, while we can use simple structural metrics such as size and complexity to identify *God Class* and *Spaghetti Code* instances, the ML model using structural information does not include precise metrics describing other aspects such as laziness or intimacy level between classes.

**Table 12:** Aggregate results reporting the comparison of the warning-based model with the metric-based one

	Warning				Metric			
	Prec.	Recall	FM	MCC	Prec.	Recall	FM	MCC
God Class	0.49	0.47	0.48	0.48	0.30	0.83	0.44	0.49
Complex Class	0.34	0.34	0.34	0.34	0.18	0.61	0.27	0.32
Spaghetti Code	0.31	0.19	0.24	0.24	0.15	0.34	0.21	0.22
Inappropriate Intimacy	0.21	0.15	0.17	0.17	0.10	0.23	0.14	0.15
Lazy Class	0.17	0.12	0.14	0.14	0.00	0.00	0.00	0.00
Middle Man	0.56	0.07	0.13	0.20	0.00	0.00	0.00	0.00
Refused Bequest	0.39	0.09	0.15	0.18	0.21	0.02	0.03	0.06

**Fig. 7:**
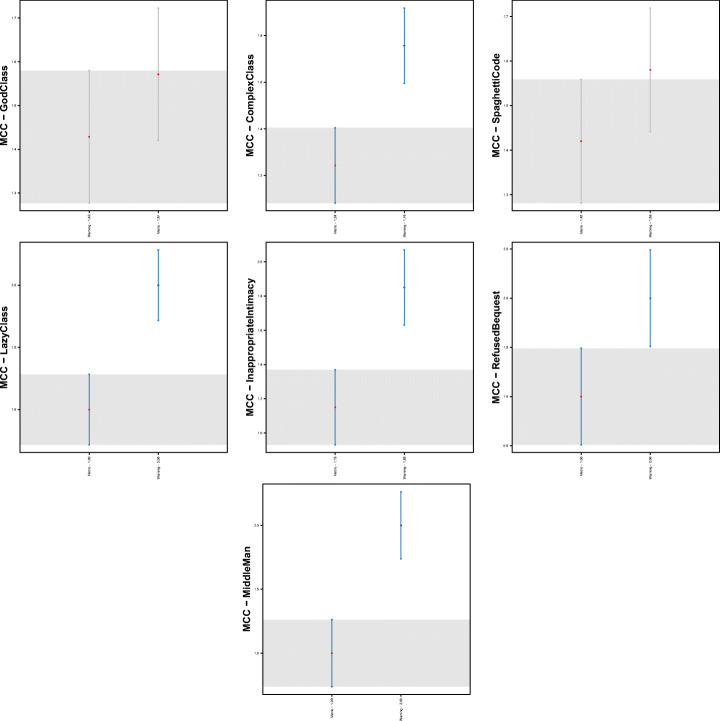
Plots representing the results of Nemenyi test for statistical significance between the MCC values obtained by Random Forest trained on static analysis warnings and structural metrics for code smells detection

The results of the Nemenyi test depicted in Fig. 7, confirm that in the cases described above there is a statistically significant difference in the two distributions. On the other hand, with respect to *God Class*, and *Spaghetti Code* it is not possible to clearly establish which of the models perform better. 
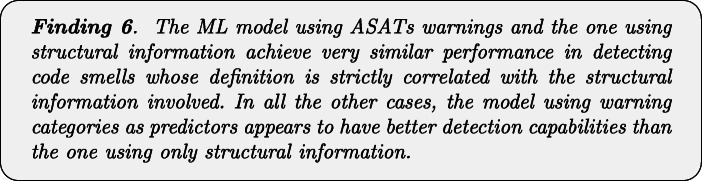

### RQ_7_. Orthogonality between the warning- and metric-based Prediction Models

Table 13 reports results of the complementarity analysis conducted between the warning- and the metric-based machine learning prediction models. The most evident result is that, regardless of the code smell considered, the two techniques show a strong overlap, i.e., most of the smelly instances identified by a technique are also identified by the other. Such a strong overlap could indicate that using metrics and warnings in combination would not lead to performance improvements. This is particularly true for *Lazy Class*, *Refused Bequest*, and *Middle Man* for which there is a very small complementarity. However, as for *God Class*, *Complex Class*, *Spaghetti Code*, and *Inappropriate Intimacy*, results show that there exist a number of smelly instances that only one of the techniques is able to detect, thus indicating a complementarity, even if limited. Therefore, it could be still worth to assess the performance achieved by a machine learner based on both warnings and structural metrics. 
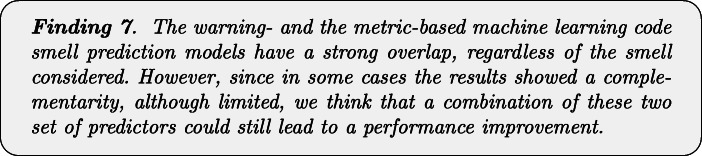

**Table 13:** Overlap analysis between the warning- and metric-based Prediction Models

Code Smell	Warning ∩ Metric	Warning ∖ Metric	Metric ∖ Warning
God Class	81%	11%	6%
Complex Class	76%	16%	8%
Spaghetti Code	72%	18%	10%
Inappropriate Intimacy	64%	22%	22%
Lazy Class	98%	1%	1%
Middle Man	86%	9%	5%
Refused Bequest	89%	7%	4%

### RQ_8_. Combining Static Analysis Warnings and Code Metrics

**Fig. 8:**
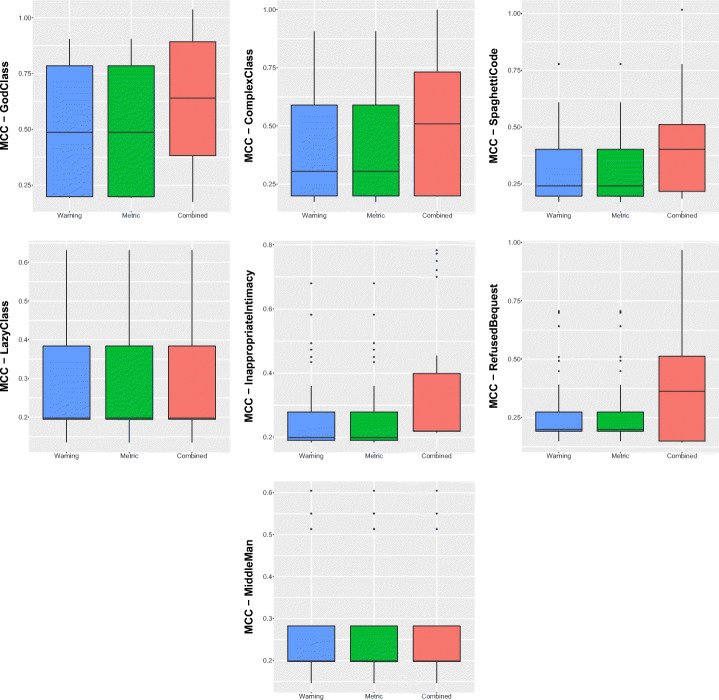
Boxplots representing the MCC values obtained by Random Forest trained on static analysis warnings and on the combination of static analysis warnings with structural metrics for code smells detection

Table 14 and Fig. 8 report the results of the performance achieved by the two model based only on ASATs warnings and code metrics, and the one combining warnings and structural information. Regardless of the considered code smell type, the full model, i.e., the one considering both warnings and structural metrics, appears to slightly outperform the other two. This is particularly true for *God Class*, *Complex Class*, *Spaghetti Code*, and *Inappropriate Intimacy*.

**Table 14:** Aggregate results reporting the comparison of the combined model with the model combining warnings categories and structural metrics

	Warning				Metric				Combined			
	Prec.	Recall	FM	MCC	Prec.	Recall	FM	MCC	Prec.	Recall	FM	MCC
God Class	0.49	0.47	0.48	0.48	0.30	0.83	0.44	0.49	0.53	0.58	0.56	0.55
Complex Class	0.34	0.34	0.34	0.34	0.18	0.61	0.27	0.32	0.39	0.43	0.41	0.41
Spaghetti Code	0.31	0.19	0.24	0.24	0.15	0.34	0.21	0.22	0.36	0.21	0.25	0.27
Inappropriate Intimacy	0.21	0.15	0.17	0.17	0.10	0.23	0.14	0.15	0.08	0.09	0.10	0.11
Lazy Class	0.17	0.12	0.14	0.14	0.00	0.00	0.00	0.00	0.19	0.12	0.15	0.15
Middle Man	0.56	0.07	0.13	0.20	0.00	0.00	0.00	0.00	0.17	0.06	0.10	0.13
Refused Bequest	0.39	0.09	0.15	0.18	0.21	0.02	0.03	0.06	0.34	0.14	0.20	0.21

**Fig. 9:**
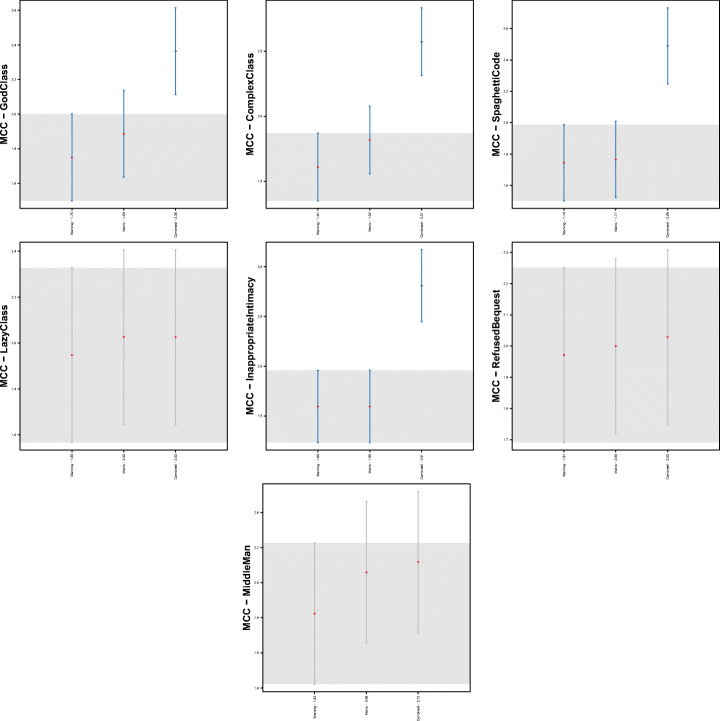
Plots representing the results of Nemenyi test for statistical significance between the MCC values obtained by Random Forest trained on static analysis warnings and on the combination of static analysis warnings with structural metrics for code smells detection

Nemenyi test results, reported in Fig. 9, confirm that for *God Class*, *Complex Class*, and *Inappropriate Intimacy* the full model performs significantly better than the others. This result is in line with **RQ**_7_ findings. Indeed, a higher complementarity has been shown for such smells, therefore the combined model is able to significantly improve the performance of warning- and metric-based machine learners.

The reported results clearly indicate that adding more information to ML classifiers helps to improve the overall performance in most cases. However, on the other hand, there is still the need of defining a set of metrics that could further improve code smell detection techniques’ performance. Our suggestion for future studies is to involve a wider set of predictors of various kinds (e.g., structural, textual, historical) in order to give the classifiers as much information as possible. 
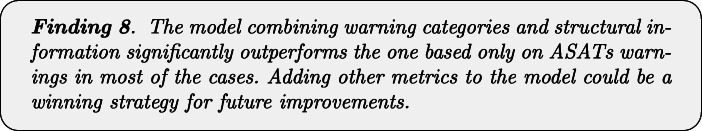


## Discussion and Implications of the Study

The results of the study pointed out a number of findings and implications for researchers that deserve further discussion.

**On the implications of the performance achieved.**: The results of our analyses have shown that a combination of features can improve the performance of ML-based code smell detection. This was true when combining static analysis warnings raised by different automated tools, but also when combining the warnings with code metrics considered by previous work. But is this enough? To further understand this point, we have compared the performance of the proposed combined model with those of three baselines: (i) the Optimistic Constant classifier, that classifies any instance as smelly; (ii) the Pessimistic Constant classifier, that classifies any instance as non-smelly; and (iii) a Random classifier, which classifies an instance as smelly or non-smelly with a probability of 50%. We performed this comparison in terms of Type I, that counts the number of false positive errors, and Type II, that counts the number of false negative errors. The selection of these two metrics was inspired by previous work in the literature (Haiduc et al. 2012). Table 15 reports the total number of Type I and Type II errors. Results show that, regardless on the code smell under consideration, the Pessimistic Constant achieves the best results in terms of total errors, i.e., Type I + Type II, thus pointing out once again the low performance of ML-based code smell detection techniques.

**Table 15:** Type I and Type II Errors Achieved in the comparison between the combined model, the optimistic constant, the pessimistic constant, and a random classifier

	Combined model		Optimistic Constant		Pessimistic Constant		Random	
Code Smell	Type I	Type II	Type I	Type II	Type I	Type II	Type I	Type II
God Class	4034 (4.68%)	214 (0.25%)	85799 (99.53%)	0 (0.00%)	0 (0.00%)	403 (0.47%)	43156.5 (50.06%)	650.5 (0.75%)
Complex Class	4907 (7.15%)	183 (0.27%)	68375 (99.60%)	0 (0.00%)	0 (0.00%)	277 (0.40%)	34372.5 (50.07%)	26.5 (0.04%)
Spaghetti Code	5005 (5.71%)	669 (0.76%)	86886 (99.09%)	0 (0.00%)	0 (0.00%)	796 (0.91%)	44526 (50.78%)	391.5 (0.45%)
Inappropriate Intimacy	728 (1.10%)	175 (0.26%)	65879 (99.69%)	0 (0.00%)	0 (0.00%)	205 (0.31%)	33984 (51.43%)	1202.5 (1.82%)
Lazy Class	1698 (3.29%)	108 (0.21%)	51525 (99.76%)	0 (0.00%)	0 (0.00%)	123 (0.24%)	26419.5 (51.15%)	101.5 (0.20%)
Middle Man	3695 (9.10%)	62 (0.15%)	40537 (99.83%)	0 (0.00%)	0 (0.00%)	70 (0.17%)	21271.5 (52.38%)	221.5 (0.55%)
Refused Bequest	8837 (11.28%)	377 (0.48%)	77870 (99.40%)	0 (0.00%)	0 (0.00%)	467 (0.60%)	37824.5 (48.28%)	1698.5 (2.17%)
  These results lead to clear implications: The problem of code smell detection through machine learning still requires specific features that have not been taken into account yet. Moreover, additional AI-specific instruments should be considered in the future with the aim of improving the code smell detection capabilities of these techniques.
**On static analysis warnings and code smells.**: According to the results of **RQ**_2_, the gain provided by the warnings raised by static analysis tools to the predictions done when using those warnings as features for code smell detection is limited. These results revealed a limited connection between the types of issues raised by static analysis tools and the specific code smells considered in the study. While this poor connection might be due to the fact that static analysis tools aim at capturing a wider set of general source code issues, we still claim that our results are somehow worrisome since they show that the warnings given to developers do not evidently refer to any design problem that previous research has related to change- and fault-proneness (Khomh et al. 2012; Palomba et al. 2018a). To some extent, such a low relation with code smells might be one of the causes leading developers to ignore the warnings raised by static analysis tools in practice (Emanuelsson and Nilsson 2008; Vassallo et al. 2019). On the one hand, our findings suggest that further studies on the relation between static analysis tools and code smells should be performed. On the other hand, tool vendors could exploit the reported results in order to propose some tuning of the static analysis tools that enable the identification of code smell-related warnings.
**A possible factor influencing the performance.**: As a complementary and follow-up discussion, our analyses conducted in **RQ**_4_ revealed that classification models built using static analysis warnings have a very low precision. While in the context of the paper we mainly highlighted the poor precision from the perspective of the models, and given for granted the poor relation between static analysis warnings and code smells discussed above, another problem might have been the cause of our results: the amount of false positive warnings raised by static analysis tools. While we did not establish the amount of false positives output by the static analysis tools in our context, this is a well-known problem that has been raised in literature (Johnson et al. 2013) and that, very likely, has had some influence on our findings. On the one hand, we plan to further investigate this aspect and possibly quantify the influence of false positives on our results. On the other hand we can still remark, for the benefit of researchers working in this field, that the problem of false positives is something that might have impacted the overall contribution that static analysis tools may have provided to the experimented code smell detection models. As such, our results might be seen as an additional motivation to investigate novel instruments to improve current static analysis tools.
**On the connection with the state of the art.**: The empirical studies conducted in this paper represented the first attempt to make static analysis warnings useful for code smell detection. Unfortunately, the results achieved confirmed the current knowledge on the state of machine learning-based code smell detection. At the same time, our findings extend the body of knowledge under two perspectives. First, researchers in the field of code smells might take advantage of our study to further investigate the reasons behind our results, possibly revealing the causes leading static analysis warnings to be not effective for detecting code smells or even proposing alternative solutions to make them work. Second, researchers in the field of automated static analysis might be interested in understanding the reasons why currently available tools do not properly support the identification of diffused and dangerous design issues, even tough certain specific warnings types are supposed to provide indications in this respect.
**Large-scale experimentations matter.**: With respect to the preliminary findings achieved in our previous work (Lujan et al. 2020a), our new results did not confirm the suitability of static analysis warnings for the detection of code smells through machine learning methods. This was due to the larger-scale nature of this experiment, where we tested the devised approaches on a dataset containing 20 more projects than the preliminary study. Therefore, as a meta-result our analyses confirmed the importance of large-scale experimentations in software engineering as a way to draw more definitive conclusions on a phenomenon of interest. Hence, based on our experience, we can recommend researchers to carefully consider the scale of the experiments when running empirical studies and take into account the overall generalizability of the reported findings when reporting and discussing results.

## Threats to Validity

Some aspects might have threaten the validity of the results achieved in our empirical study. This section reports on these aspects and explains how we mitigated them, following the guidelines provided by Wohlin et al. (2000).

### Construct Validity

Threats in this category concern with the relationship between theory and observation. These are mainly due to possible measurement errors. A first discussion point is related to the dataset exploited in our study. In this respect, we decided to rely on a dataset reporting manually-validated code smell instances: this decision was based on previous findings showing that the meaningfulness and actionability of the results highly degrade when considering tool-based oracles (Di Nucci et al. 2018a). As such, our choice made the findings more reliable—we did not include in our ground-truth false positives and negatives—at the cost of having less systems analyzed: we are aware of this possible limitation and we plan indeed to conduct larger-scale analyses as part of our future research agenda.

When it comes to the selection of the automated static analysis tools, we considered three of the most reliable and adopted tools (Vassallo et al. 2019). Nevertheless, we cannot exclude the presence of false positives or false negatives in the detected warnings. While this may have influenced the results achieved, our study showed that the performance of code smell prediction models can be fairly high even in presence of false positives and negatives: this means that, in cases of tools giving a lower amount of false alarms or being able to provide more correct information, the accuracy of the proposed learners might be even increased. In any case, further analyses targeting the impact of misinformation on the performance of the learners are part of our future research agenda.

### Internal Validity

These threats are related to the internal factors of the study that might have affected the results. When assessing the role of static analysis tools for code smell detection, we took into account three tools with the aim of increasing our knowledge on the matter. Yet, we recognize that other tools might consider different, more powerful warnings that may affect the performance of the learners. Also in this case, further analyses are part of our future research agenda.

### External Validity

As for the generalizability of the results, our empirical study considered all the systems that could be actually analyzed from the exploited public dataset (Palomba et al. 2018a; Palomba et al. 2015). As also reported above, we are aware that our analyses have been bounded by technical limitations, e.g., the inability to compile some of the systems in the dataset, or by design decisions, e.g., the choice of considering a dataset containing actual code smell instances. Nonetheless, we preferred to conduct a more precise and reliable analysis, sacrificing quantity. Yet, we do believe that the results presented represent a valuable base for researchers, practitioners, and tool vendors that can be used and/or extended to reconsider the role of static analysis tools in the context of software quality assessment and improvement. In this respect, we also highlight the need for additional publicly available datasets of validated code smell instances, which might allow more generalizable and reliable investigations.

### Conclusion Validity

These threats are related to the relationship between the treatment and the outcome. In our research, we adopted different machine learning techniques to reduce the bias of the low prediction power that a single classifier could have. In addition, we did not limit ourselves to the usage of these classifiers, but also addressed some of the possible issues arising when employing them. For instance, we dealt with multicollinearity problems, hyper-parameter configuration, and data unbalance. We recognize, however, that other statistical or machine learning techniques (e.g. deep learning) might have yielded similar or better accuracy than the techniques we used.

Last but not least, we applied the Nemenyi test (Nemenyi 1962) to statistically verify the performance achieved by the experimented machine learning approaches.

## Conclusion

In this paper, we assessed the adequacy of static analysis warnings in the context of code smell prediction. We started by analyzing the contribution given by each warning type to the prediction of seven code smell types. Then, we measured the performance of machine learning models using static analysis warnings as features and aiming at identifying the presence of code smells.

The results achieved when experimenting the individual models revealed low performance: this was mainly due to their poor precision. In an effort of dealing with such low performance, we considered the possibility to combine the warnings raised by different static analysis tools: in this regard, we first measured the orthogonality of the code smell instances correctly identified by machine learners exploiting different warnings; then, we combined these warnings in a combined model.

The results of our study reported that, while a combined model can significantly improve the performance of the individual models, it yields a similar accuracy than the one of a random classifier. We also found out that machine learning models built using static analysis warnings reach a particularly low accuracy when considering code smells targeting coupling and inheritance properties of source code. The outcomes of this empirical study represent the main inputs for our future research agenda, which is mainly oriented to face the challenges related to the definition of ad-hoc features for code smell detection through machine learning approaches. In addition, part of our future research work in the area will be devoted to the *qualitative* analysis of the role of static analysis warnings for code smell detection. In particular, we plan to complement the achieved findings through investigations conducted on source code snippets mined from StackOverflow, for which we plan to analyze the relation between the posts issued by developers and related to static analysis warnings and the presence of code smells in those snippets. We also plan to extend the scope of our work with method-level code smells. In this respect, we aim at defining the most appropriate tools and data analysis methodologies that may help investigating how static analysis warnings impact the detection of this category of code smells. Last but not least, we plan to systematically assess deep learning methods (Das et al. 2019; Liu et al. 2019), which might more naturally combine features, given that they act directly on source code.

## Footnotes

1 The FindBugs official documentation: http://findbugs.sourceforge.net/bugDescriptions.html.

2 https://checkstyle.sourceforge.io

3 http://findbugs.sourceforge.net/findbugs2.html

4 https://pmd.github.io/latest/

5 https://checkstyle.org/#Download

6 https://github.com/checkstyle/checkstyle/tree/master/src/main/resources

7 https://github.com/pmd/pmd/releases/download/pmd_releases%2F6.23.0/pmd-bin-6.23.0.zip

8 https://github.com/pmd/pmd/tree/master/pmd-java/src/main/resources/rulesets/java
